# The early antibiotic therapy in septic patients - milestone or sticking point?

**DOI:** 10.1186/s13054-014-0671-1

**Published:** 2014-11-30

**Authors:** Michael Bernhard, Christoph Lichtenstern, Christian Eckmann, Markus A Weigand

**Affiliations:** Emergency Department, University Hospital of Leipzig, Liebigstrasse 20, D-04103 Leipzig, Germany; Department of Anesthesiology, University Hospital of Heidelberg, Im Neuenheimer Feld 110, D-69120 Heidelberg, Germany; Department of General, Visceral and Thoracic Surgery, Academic Hospital of Medical University Hannover, Virchowstrasse 8h, D-31221 Peine, Germany

## Abstract

Sepsis is one of the oldest and most elusive syndromes in medicine. Every effort should be made to treat these patients with the best available evidence. As a milestone, empiric antimicrobial therapy is essential in order to reduce morbidity and mortality of septic patients. As a sticking point, the use of broad-spectrum antimicrobial agents may be associated with induction of resistance among common pathogens.

## Rationale for early antibiotic therapy

Worldwide, the annual prevalence of sepsis is estimated at 19 million cases [1]. The mortality rates in severe sepsis were reduced to 20% to 30% because of advances in training, recognition, surveillance, monitoring, and rapid initial antibiotic therapy and organ support [1,2]. The most recent update of the Surviving Sepsis Campaign guidelines was published in 2013 [3]. A consensus committee provided valuable and clear recommendations on treatment of sepsis and septic shock (Table 1). One of the main focuses is the administration of a broad-spectrum antibiotic (Table 2) [3]. It is recommended that antimicrobials be administrated within the first hour of recognition of septic shock and severe sepsis without septic shock [4–7]. Moreover, it is recommended that initial anti-infective therapy include one or more drugs that have activity against all likely pathogens and penetrate in adequate concentrations into the target tissue [3]. The selected antibiotic strategy should anticipate the site of infection, medical and culture history, and local microbial susceptibility results, all in an emergency situation [1,8].

**Table 1:** **Extract of the key recommendations and suggestions of the Surviving Sepsis Campaign guidelines** [3]

●	Early quantitative resuscitation of the septic patient during the first 6 hours after recognition (1C)
●	Blood cultures before antibiotic therapy (1C)
●	Imaging studies performed promptly to confirm a potential source of infection (UG)
●	Administration of broad-spectrum antimicrobials therapy within 1 hour of the recognition of septic shock (1B) and severe sepsis without septic shock (1C) as the goal of therapy
●	Reassessment of antimicrobial therapy daily for de-escalation, when appropriate (1B)
●	Infection source control with attention to the balance of risks and benefits of the chosen method within 12 hours of diagnosis (1C)

Principles of the Grading of the Recommendations Assessment, Development, and Evaluation (GRADE) system to guide assessment of quality of evidence from high (A) to very low (D) and to determine the strength of recommendations as strong (1) or weak (2). UG, ungraded.

**Table 2:** **Extract from the Surviving Sepsis Care bundles** [3]

To be completed within 3 hours	
1.	Measure lactate levels.
2.	Obtain blood cultures prior to administration of antibiotics.
3.	Administer broad-spectrum antibiotics.
4.	Administer 30 mL/kg crystalloid for hypotension of lactate of at least 4 mmol/L.

## New research results

### Appropriate antibiotic therapy

In a systematic review and meta-analysis, Paul and colleagues [9] found a pooled odds ratio of appropriate antibiotic therapy during the first 48 hours for all-cause mortality of 1.60 (95% CI 1.37 to 1.86), corresponding to a number needed to treat of 10 (95% CI 8 to 15). Kumar and colleagues [4] found a time-dependent mortality with a 7.6% decrease in survival for each hour of antibiotic delay in patients with sepsis.

### Timing of antimicrobial therapy

Some recently published investigations supported the findings by Kumar and colleagues [4] (Table 3). Ferrer and colleagues [10] presented a retrospective analysis of a large dataset collected prospectively. In total, 28,150 patients with severe sepsis and septic shock from 165 ICUs in Europe, the US, and South America were included. Of them, 17,990 patients received antibiotics after identification of sepsis, and the in-hospital mortality was 29.7%. After 1 hour, hospital mortality steadily increased with a delay in antibiotic timing. The adjusted hospital mortality odds ratios steadily increased from 1.00 to 1.52 as time to antibiotic administration increased from 0 to greater than 6 hours where 0 to 1 hour was the reference group. The probability of mortality increased from 24.6% to 33.1% (*P* <0.001) [10]. However, critics stated that, owing to a lack of information on antibiotic appropriateness and focus control, this study was limited [1].

**Table 3:** **Comparison of studies investigating antibiotic treatment in patients with sepsis**

**Parameter**	**Kumar** ***et al*** **. [** 4 **]** ***Crit Care Med*** **(2006)**	**Ferrer** ***et al*** **. [** 10] ***Crit Care Med*** **(2014)**	**Puskarich** ***et al*** **. [** 11 **]** ***Crit Care Med*** **(2011)**	**Bloos** ***et al*** **. [** 12 **]** ***Crit Care*** **(2014)**	**Hranjec** ***et al*** **. [** 13 **]** ***Lancet Infect Dis*** **(2012)**	
**Study design**	**Retrospective multicenter cohort study**	**Retrospective analysis of prospective collected dataset multicenter**	**Prospective preplanned analysis of a multicenter randomized clinical trial**	**Prospective multicenter cohort study**	**Prospective quasi-experimental, before-and-after observational study single center**	
**Setting**	**ICU septic shock**	**ICU mixed**	**ED septic shock**	**ICU**	**ICU-acquired infection**	
					**Aggressive**	**Conservative**
Patients	2,731	17,993	291	1,011	247	237
Age, years	63	NR	62 (IQR 50–73)	69	NR	NR
Gender, male	54%	NR	53%	63%	NR	NR
APACHE score	26 ± 9	NR	NR	NR	NR	NR
SAPS II	NR	NR	42 (IQR 30–55)	48 (IQR 37–60)	NR	NR
SOFA score	NR	NR	6 (IQR 4–9)	10 (IQR 8–12)	NR	NR
MEDS score	NR	NR	11 (IQR 8–14)	NR	NR	NR
Septic shock	2,731 (100%)	11,558 (64.2%)	291 (100%)	NR	38.5%	46.4%
Positive BC	34.2%	NR	100 (34.4%)	317 (48.8%)	NR	NR
Nosocomial	58.1%	12.2%	NR	56.2%	NR	NR
Median time to shock recognition	NR	NR	89 (IQR 48–180)	NR	NR	NR
Overall mortality	56.2%	31.3%	55 (18.9%)	41.4%	99 (40.1%)	50 (21.1%)
Mortality for BC-positive septic shock	NR	NR	26/100 (26.0%)	NR	NR	NR
Mortality for BC-negative septic shock	NR	NR	29/191 (15.2%)	NR	NR	NR
Infection site						
Pneumonia	1,016 (37.2%)	8,487 (47.2%)	99 (34.0%)	351 (34.9%)	75 (20%)	93 (39%)
UTI	293 (10.7%)	4,757 (26.4%)	71 (24.4%)	122 (12.1%)	33 (13%)	36 (15%)
Intra-abdominal	801 (29.3%)	3,505 (19.5%)	49 (16.8%)	366 (36.3%)	31 (13%)	22 (9%)
Skin and soft tissue	197 (7.2%)	1,133 (6.3%)	23 (7.9%)	NR	NR	NR
Intravascular catheter	100 (3.7%)	661 (3.7%)	11 (3.8%)	NR	14 (6%)	8 (3%)
Surgical wounds	31 (1.1%)	815 (4.5%)	7 (2.4%)	NR	19 (8%)	21 (9%)
Endocarditis	NR	187 (1.0%)	4 (1.4%)	NR	NR	NR
CNSI (e.g., meningitis)	20 (0.7%)	277 (15%)	3 (1.0%)	NR	NR	NR
Septic arthritis	21 (0.8%)	NR	2 (0.7%)	NR	NR	NR
SDI	58 (2.1%)	NR	1 (0.3%)	NR	NR	NR
Ear, nose, throat	NR	NR	1 (0.3%)	NR	NR	NR
Toxic shock syndrome	NR	NR	1 (0.3%)	NR	NR	NR
Unknown	120 (4.4%)	NR	40 (13.8%)	50 (5%)	49 (20%)	46 (19%)
Two or more sources	NR	NR	21 (7.2%)	NR	NR	NR
Mediastinitis	15 (0.5%)	NR	NR	NR	NR	NR
Other	59 (2.1%)	1,980 (11.0%)	NR	105 (10.4%)	26 (11%)	11 (5%)
Bone	NR	232 (1.3%)	NR	NR	NR	NR
Device	NR	219 (1.2%)	NR	NR	NR	NR
Bone/soft tissue	NR	NR	NR	72 (7.1%)	NR	NR
Upper airway	NR	NR	NR	83 (8.2%)	NR	NR

APACHE, Acute Physiology and Chronic Health Evaluation; BC, blood culture; CNSI, central nervous system infection; ED, emergency department; IQR, interquartile range; MEDS, Mortality in Emergency Department Sepsis; NR, not reported; SAPS II, Simplified Acute Physiology Score II; SDI, systemically disseminated infection; SOFA, Sequential Organ Failure Assessment; UTI, urinary tract infection.

In contrast, Puskarich and colleagues [11] reported results from a large prospective study of emergency department patients with septic shock, which failed to demonstrate an association between timing of antibiotic administration from emergency department triage and hospital mortality. A delay in antibiotics until after shock recognition, as compared with before, was associated with increased mortality; however, if antibiotics are administered after shock recognition, there is no increase in mortality with hourly delays. These findings were in contrast to those from Kumar and colleagues [4] and Ferrer and colleagues [10]. The differences may be explained by a higher severity of illness in the other two studies. For example, Kumar and colleagues [4] investigated ICU patients with septic shock with an overall mortality of 56% in comparison with the emergency department patients in the study by Puskarich and colleagues [11] with 19%. With respect to these findings, the focus on the observed patient cohort seems to be essential.

In a prospective observational multicenter cohort study in 44 German ICUs including 1,011 patients with severe sepsis and septic shock, Bloos and colleagues [12] did not find a linear association between timing of antibiotic therapy and 28-day mortality. However, regardless of timing, 28-day mortality rate was lower in patients with adequate antibiotic therapy than in those with non-adequate antibiotic therapy (30% versus 41%, *P* <0.001). Bloos and colleagues stated that, owing to differences in the related patient populations, they were not able to confirm the findings of Kumar and colleagues [4].

Given these findings, the concept of early empiric antibiotic therapy has recently been challenged. More and more, the underlying and treated patient population has come into focus.

## Escalating versus de-escalating strategy

With respect to the previously reported investigations, Hranjec and colleagues [13] presented data from a 2-year, quasi-experimental before-and-after observational study of hemodynamically stable patients admitted to a surgical ICU. In the first year, patients suspected of having an infection (n =101, aggressive approach, de-escalating strategy) had blood cultures and antimicrobial therapy was started. In the second year, patients suspected of having an infection (n =100, conservative approach, escalating strategy) had an antimicrobial therapy only after objective findings confirmed an infection. The conservative approach was associated with lower all-cause mortality: (13/100) 13% versus (27/101) 27%, *P* =0.015. The odds ratio for the risk of mortality in the aggressive approach group was 2.5 (95% CI 1.5 to 4.0) in comparison with the conservative group. In this investigation, waiting for objective data to diagnose infection before treatment with antimicrobial agents for suspected infection does not worsen the mortality.

## Conclusions

Early broad-spectrum antimicrobial therapy is necessary within the ‘golden hour’ in septic shock and, as a milestone, reduces mortality. However, the use of broad-spectrum antimicrobial agents may be associated with induction of resistance among common pathogens and therefore may be a sticking point. In septic patients without sepsis-associated hypotension, diagnostic measure may be beneficial before an antibiotic therapy starts. New research is urgently needed concerning different strategies that would balance early administration of antibiotics against the potential harmful effects to patients and resistance. New research strategies have to test a de-escalating strategy with restriction of specific broad-spectrum antibiotics in the initial therapy to the most critically ill patients and patients with suspicion of multidrug-resistant pathogens, against an escalating strategy in less critically ill patients. Moreover, further studies are needed to distinguish between more and less critically ill patients, to differentiate patients’ backgrounds, and to determine indicators for patients who profit from an escalating or de-escalating strategy.
